# Estimated Rates of Retinal Ganglion Cell Loss in Glaucomatous Eyes with and without Optic Disc Hemorrhages

**DOI:** 10.1371/journal.pone.0105611

**Published:** 2014-08-26

**Authors:** Carolina P. B. Gracitelli, Andrew J. Tatham, Linda M. Zangwill, Robert N. Weinreb, Ting Liu, Felipe A. Medeiros

**Affiliations:** 1 Hamilton Glaucoma Center and Department of Ophthalmology, University of California San Diego, San Diego, California, United States of America; 2 Department of Ophthalmology, Federal University of São Paulo, São Paulo, São Paulo Brazil; 3 Princess Alexandra Eye Pavilion and Department of Ophthalmology, University of Edinburgh, Edinburgh, United Kingdom; Instituto Murciano de Investigación Biosanitaria-Virgen de la Arrixaca, Spain

## Abstract

**Purpose:**

To evaluate whether optic disc hemorrhages are associated with faster rates of estimated retinal ganglion cell (RGC) loss in glaucoma.

**Methods:**

A longitudinal observational cohort study of 222 eyes of 122 patients with glaucoma recruited from the Diagnostic Innovations Glaucoma Study (DIGS) followed for an average of 3.74±0.85 years. All subjects had optical coherence tomography and standard automated perimetry during follow up. Optic disc hemorrhages were detected by masked evaluation of stereophotographs. Rates of change in estimated numbers of RGCs were determined using a previously described method. A random coefficients model was used to investigate the relationship between disc hemorrhages and rates of change in estimated RGC counts over time.

**Results:**

19 eyes of 18 subjects had at least one disc hemorrhage during follow up. At baseline, average estimated RGC counts in eyes with and without disc hemorrhages were 677,994 cells and 682,021 cells, respectively (P = 0.929). Eyes with optic disc hemorrhages during follow-up had significantly faster rates of estimated RGC loss than eyes without disc hemorrhages (22,233 cells/year versus 10,704 cells/year, P = 0.020). The effect of disc hemorrhages on the rates of estimated RGC loss remained significant after adjusting for confounding variables.

**Conclusion:**

Eyes with disc hemorrhages showed faster rates of RGC loss compared to eyes without disc hemorrhages. These results provide further evidence that disc hemorrhages should be considered as an indicator of increased risk for faster neural loss in glaucoma.

## Introduction

Glaucoma is an optic neuropathy characterized by progressive loss of retinal ganglion cells (RGCs) and associated morphological changes to the optic nerve and retinal nerve fiber layer (RNFL).[1] Visual field loss from glaucoma is irreversible and, therefore, it is paramount to identify eyes at high risk of progression. Prospective studies have shown optic disc hemorrhages to be an important risk factor for the development and progression of glaucoma.[2]–[6] In this regard, patients with ocular hypertension who developed a disc hemorrhage during the course of the Ocular Hypertension Treatment Study (OHTS) had a nearly four-fold increased risk of progression to glaucoma.[7] In those with established disease, both the Early Manifest Glaucoma Trial (EMGT) and the Collaborative Normal Tension Glaucoma Study have shown an increased risk of visual field progression in eyes with optic disc hemorrhages compared to those without.[6]

Recently, Medeiros and colleagues[8] evaluated rates of visual field progression in eyes with and without optic disc hemorrhages. Eyes with disc hemorrhages were found to have significantly faster rates of progressive visual field loss than those without disc hemorrhages, further supporting disc hemorrhages as an important risk marker for progression. De Moraes et al[9] also showed that disc hemorrhages may be associated with rapid and localized visual field progression.

Although optic disc hemorrhages are widely appreciated as a risk factor for progressive glaucomatous visual field loss, visual field loss ultimately reflects underlying dysfunction and loss of RGCs. However, the relationship between disc hemorrhages and rates of RGC loss has not yet been established. Although at present RGC numbers cannot be directly quantified *in vivo* in humans, it is possible to estimate RGC counts from a combination of optical coherence tomography (OCT) measurements of retinal nerve fiber layer (RNFL) thickness and standard automated perimetry (SAP) sensitivities using empirically derived formulas. [10]–[12]

The purpose of the current study was to estimate longitudinal rates of RGC loss in patients with glaucoma and to determine whether the presence of optic disc hemorrhages was associated with a faster rate of progression.

## Methods

This was a longitudinal observational cohort study involving 222 eyes of 122 participants from the Diagnostic Innovations in Glaucoma Study (DIGS), a prospective longitudinal study designed to evaluate optic nerve structure and visual function in glaucoma. The study was conducted at the Hamilton Glaucoma Center of the Department of Ophthalmology, University of California San Diego (UCSD). Methodological details have been described previously.[13] Informed consent was obtained from all participants, and the institutional review board and human subjects committee at the University of California San Diego prospectively approved all methods. All study methods adhered to the tenets of the Declaration of Helsinki for research involving human subjects and the study was conducted in accordance with the regulations of the Health Insurance Portability and Accountability Act. And all participants provided their written informed consent to participate in this study.

At each annual visit during follow-up, patients underwent a comprehensive ophthalmologic examination including review of medical history, best-corrected visual acuity, slit-lamp biomicroscopy, intraocular pressure (IOP), dilated fundoscopic examination, stereoscopic optic disc photography, Cirrus high definition OCT (HDOCT) (Carl Zeiss Meditec Inc., Dublin, CA) and SAP using the Swedish interactive threshold algorithm (SITA standard 24-2; Carl Zeiss Meditec, Inc, Dublin, Caifornia, USA). In addition, every six months IOP, SAP, HDOCT images were obtained. Central corneal thickness was measured once during follow-up using an ultrasound pachymeter (Pachette GDH 500; DGH Technology, Inc., Philadelphia, PA). Only patients with open angles on gonioscopy were included. Subjects were excluded if they had a best-corrected visual acuity of less than 20/40, spherical refraction outside±5.0 diopters, cylinder correction outside 3.0 diopters, or both; or any other ocular or systemic disease that could affect the optic nerve or the visual field.

All patients had a diagnosis of glaucoma at baseline, with glaucoma defined by the presence of repeatable (> =  3 consecutive) abnormal SAP tests. Eyes with documented evidence of progressive glaucomatous optic disc changes noted on masked grading of stereophotographs were also classified as glaucomatous, irrespective of visual field findings. SAP tests were defined as normal if the mean deviation (MD) and pattern standard deviation (PSD) were within 95% normal confidence limits and the Glaucoma Hemifield Test (GHT) was also within normal limits. An abnormal SAP test was defined as a visual field with a PSD with P <0.05 and/or a GHT outside normal limits. For the purposes of the analysis, eyes were classified into two groups based on whether or not a disc hemorrhage was detected on masked stereophotographs at any period during follow up.

### Stereophotographs

All patients had stereoscopic optic disc photographs repeated at least every 12 months during follow-up. The images were reviewed with a stereoscopic viewer (Screen- VU stereoscope; PS Manufacturing, Portland, Oregon, USA) by 2 or more experienced graders masked to the subjects' identity and to the other test results. The methodology used to grade optic disc photographs for progression at the UCSD Optic Disc Reading Center has been provided elsewhere.[13]–[15] Discrepancies between the 2 graders were resolved by consensus or adjudication by a third experienced grader. Disc hemorrhages had to be located within 1 disc diameter from the optic disc border and not associated with optic disc edema, papillitis, diabetic retinopathy, central or branch retinal vein occlusion, or any other retinal disease.

### Optical coherence tomography

The Cirrus HDOCT (software v. 5.2, Carl Zeiss Meditec Inc., Dublin, CA, model 4000) was used to measure RNFL thickness in this study. This device uses a superluminescent diode scan with a center wavelength of 840 nm and an acquisition rate of 27 000 A-scans per second at an axial resolution of 5 µm. The protocol used for RNFL thickness measurement was the optic disc cube with circumpapillary RNFL thickness measurements calculated from a 3.46-mm diameter circular scan (10.87-mm length) automatically placed around the optic disc. An experienced examiner, masked to the results of other tests, evaluated the quality of all OCT scans. Good quality scans had to have focused images from the ocular fundus, signal strength greater than 7 and presence of a centered circular ring around the optic disc. Scans were also evaluated as to the adequacy of the algorithm for detection of the RNFL. Only scans without overt algorithm failure in detecting the retinal borders were included in the study.

### Standard automated perimetry

All visual fields were evaluated by the UCSD Visual Field Assessment Center (VisFACT).[16] Visual fields with more than 33% fixation losses or false-negative errors, or more than 15% false-positive errors, were excluded. Visual fields exhibiting a learning effect (i.e., initial tests showing consistent improvement on visual field indices) were also excluded. Visual fields were further reviewed for the following artifacts: eyelid and rim artifacts, fatigue effects, inappropriate fixation, evidence that the visual field results were caused by a disease other than glaucoma and inattention.

### Estimation of retinal ganglion cell number

The estimates of RGC counts were obtained according to the model developed by Medeiros et al[11], [12], [17] based on empirical formulas derived by Harwerth et al[10] for estimating ganglion cell counts from SAP and OCT. The model uses information from structural and functional tests to derive a final estimate of the RGC count in a particular eye. The details of the model and the empirical formulas used to derive RGC counts have been described in detail in previous publications. [11], [12], [17] In brief, the initial step of the model consists in translating SAP sensitivity values into RGC counts using empirical formulas derived by experimental research in monkeys and subsequently translated to normal and glaucomatous human eyes. The following formulas were used to estimate the number of RGC somas in an area of the retina corresponding to a specific SAP test field location at eccentricity *ec* with sensitivity *s* in decibels:
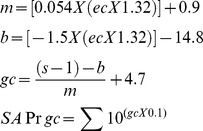



In these formulas, *m* and *b* represent the slope and intercept, respectively, of the linear function relating ganglion cell quantity (gc) in decibels to the visual field sensitivity (s) in decibels at a given eccentricity. To account for the total number of ganglion cells in an area of the retina, the cell density derived from each perimetry measurement was considered to be uniform over an area of retina corresponding to an area of 6×6 degrees of visual space that separates test locations in SAP. By applying the above formulas, a SAP-derived estimate of the total number of RGCs (SAP*rgc*) was obtained by adding the estimates from all locations in the visual field. The structural part of the model consisted in estimating the number of RGC axons from RNFL thickness measurements obtained by OCT. The model took into account the effect of aging in the axonal density and the effect of disease severity on the relationship between neuronal and non-neuronal components of the RNFL thickness estimates obtained by OCT. To derive the total number of RGC axons from the global RNFL thickness measurements obtained by OCT (OCT*rgc*), we applied the following formulas: 
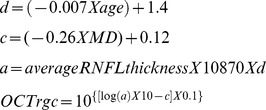



In the above formulas, *d* corresponds to the axonal density (axons/μm^2^) and *c* is a correction factor for the severity of disease to take into account remodeling of the RNFL axonal and non-axonal composition. These calculations provide an estimate of the number of RGCs from 2 sources, one functional and on structural. A combined calculation of RGC counts was performed according the following formula:
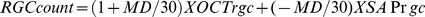



The rationale for using a weighting system for deriving the final RGC count is described by Medeiros et al, [11] [12] [17] but in essence it relies on the fact that the accuracies of clinical perimetry and imaging tests are inversely related to disease severity.

### Statistical Analysis

Descriptive statistics included mean and standard deviation and t-tests for normally distributed variables and median, interquartile range and Wilcoxon rank-sum for non-parametrically distributed variables. The evaluation of the effect of optic disc hemorrhages on rates of estimated RGC loss was performed using random coefficients models. These models are a type of linear mixed model that involves both random intercepts and random slopes, which consider the clustered structure of the data, allowing the residuals associated with the longitudinal measures on the same unit of analysis to be correlated. The details of the use of these models for evaluation of rates of change in glaucoma and to model longitudinal processes have been reported previously. [18]–[21]

The estimated number of RGCs was considered the dependent variable in the model. Disc hemorrhage (variable HEMORRHAGE) was included as a fixed-effect covariate with a value of 1 if the eye had a disc hemorrhage detected during follow up and a value of 0 if no disc hemorrhage was detected. The variable TIME (time from baseline in years) was included as a continuous predictor. The significance of the coefficients associated with the variable TIME indicated whether there is a significant trend in RGC estimates over time, i.e., whether RGC estimates tended to decrease or increase significantly over time. The two-way interaction between TIME and HEMORRHAGE (TIME x HEMORRHAGE) was included in the model to evaluate whether there was a significant difference in estimated RGC counts over time between patients with and without disc hemorrhages.

Random coefficients models were also used to evaluate the effect of possible confounding factors on the relationship between change in estimated RGC loss over time and disc hemorrhages. Variables investigated included mean IOP, CCT, baseline age, ancestry and gender. Interaction terms were included to investigate the effect of particular variables on rates of estimated RGC count change over time. To enable better interpretation of coefficients, the variables CCT, IOP and age were centered on the respective sample means.

All statistical analyses were performed with commercially available software (Stata, version 13; StataCorp LP, College Station, Texas, USA). The alpha level (type I error) was set at 0.05.

## Results

**Figure 1:**
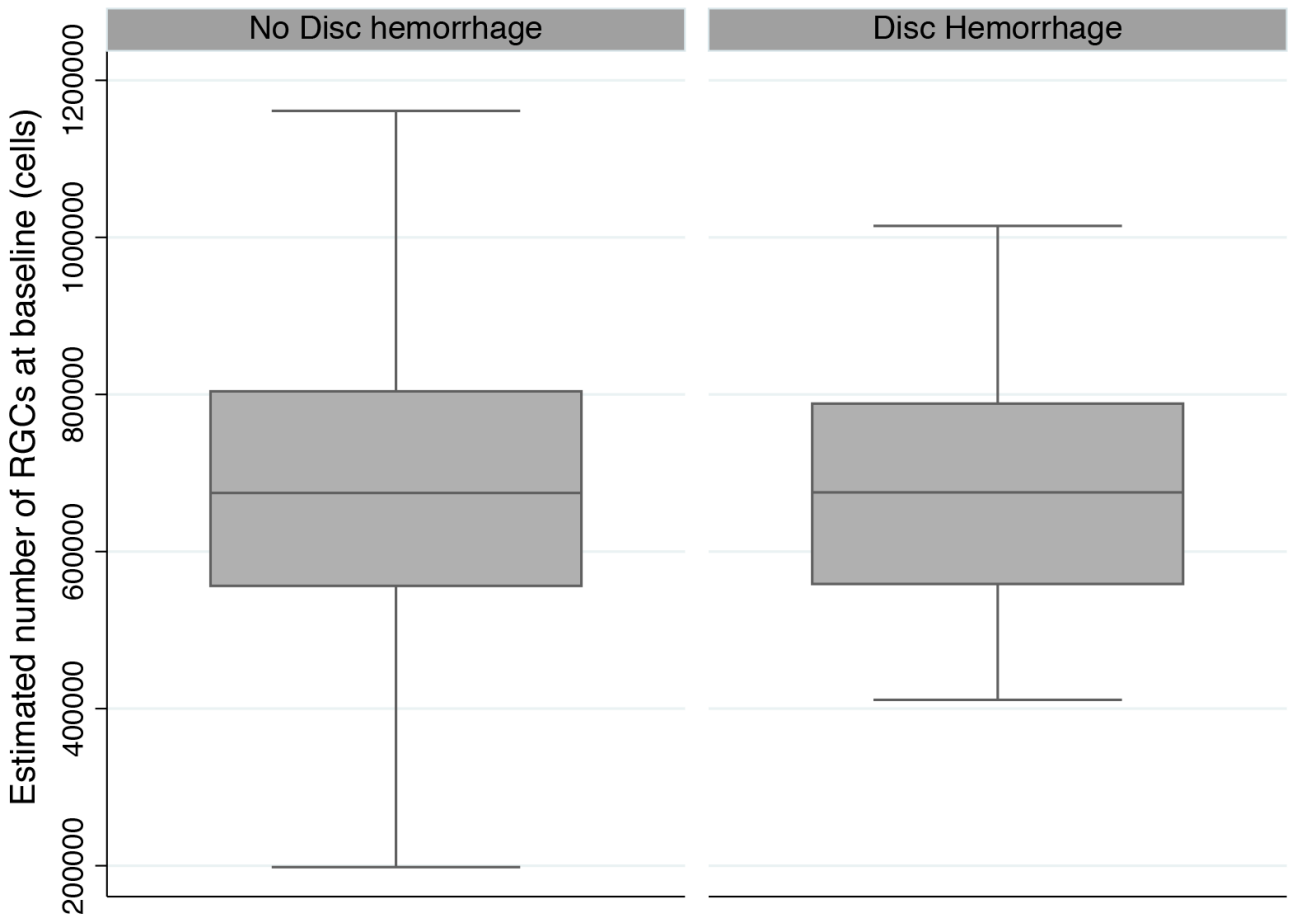
Boxplots showing the distribution of estimated number of retinal ganglion cells (RGCs) at baseline in eyes with and without optic disc hemorrhages during follow up. Box: median, and interquartile range. Boxplot with whiskers with maximum and minimum 1.5 IQR.

The study included 222 eyes of 122 participants with glaucoma followed for an average of 3.74±0.85 years. During follow up 19 of 222 eyes (8.6%) had at least one disc hemorrhage detected. 3 eyes had disc hemorrhages located in the superior region of the optic disc, 13 had inferior disc hemorrhages and 2 had both superior and inferior hemorrhages. Table 1 shows the demographic and clinical characteristics of the two groups at baseline. There was no statistically significant difference in age, ancestry or gender between those with and without disc hemorrhages. There was also no significant difference in SAP MD or average RNFL thickness at baseline for those with and without disc hemorrhages during follow up. The mean estimated number of RGCs at baseline was similar in both groups with an estimated 682,021±152,455 cells in those with disc hemorrhages compared to 677,994±196,568 cells in eyes without disc hemorrhages (P = 0.929). Figure 1 shows the distribution of estimated number of RGCs at baseline for the two groups.

**Table 1:** Demographic and clinical characteristics of the eyes included in the study.

	No Disc Hemorrhage (203 eyes, 104 patients)	Disc Hemorrhage (19 eyes, 18 patients)	*P* value
**Age, years** a	68.6±10.7	71.4±11.4	0.309
**Gender**			0.567
**Female**	56 (46%)	11 (9%)	
**Male**	48 (39%)	7 (6%)	
**Ancestry**			0.196
**European**	69 (57%)	14 (11%)	
**African**	29 (24%)	2 (1.5%)	
**Other**	6 (5%)	2 (1.5%)	
**Baseline MD, dB** b	−2.7 (−1.69)	−1.8 (−1.72)	0.415
	(−3.53 to −0.36)	(−3.15 to −0.33)	
**Baseline VFI, %** b	93.5 (98)	96.1 (98.5)	0.633
	(93 to 99)	(95.5 to 99)	
**Baseline RNFL thickness, µm** a	77.9±12.7	78.8±12.4	0.970
**Baseline estimated RGC count** a	677,994±196,568	682,021±152,455	0.929
**Baseline IOP, mmHg**	15.5±4.6	15.6±4.5	0.981
**Mean IOP, mmHg** a	14.9±4.7	15.9±4.3	0.097
**CCT, µm** a	545.4±40.3	542.8±44.7	0.968
**Follow up, years** a	3.7±0.8	3.9±0.9	0.087

Legend:

dB: decibels; MD = mean deviation, VFI = visual field index, Baseline estimated RGC count = estimated number of retinal ganglion cells at baseline (when TIME = 0); RNFL = retinal nerve fiber layer, IOP = intraocular pressure; CCT = central cornea thickness

a Mean±SD

b Mean, (median), interquartile range

Table 2 shows the results of the random coefficients model investigating the relationship between estimated RGC counts over time in patients with and without disc hemorrhages. On average, eyes with and without disc hemorrhages lost RGCs during follow-up, however, eyes with disc hemorrhages had a faster rate of loss. This is evident in the model from the significant value (P = 0.020) of the interaction term (HEMORRHAGE X TIME). The variable HEMORRHAGE was not statistically significant in the model, indicating that there was no significant difference in estimated RGC counts at baseline in eyes with and without disc hemorrhages. The interaction term (HEMORRHAGE X TIME) indicates the effect of the presence of disc hemorrhage on slopes of change in estimated RGC counts over TIME (i.e., change in estimated RGC counts during follow up). As the absence of a disc hemorrhage was used as the reference category (no hemorrhage = 0), the coefficient of the variable TIME indicates the rate of loss in the eyes without a disc hemorrhage detected during follow up. The mean estimated rate of RGC loss in the group without disc hemorrhages was 10,704 cells per year. The estimated rate of RGC loss in the group with disc hemorrhages was obtained by adding the coefficient for TIME (−10,704 cells per year) to that of the interaction term HEMORRHAGE X TIME (−11,529 cells per year), which resulted in an estimated loss of 22,233 cells per year.

**Table 2:** Results of the random coefficients model examining the association between rates of change in estimated number of retinal ganglion cells (RGCs) and the presence or absence of disc hemorrhages during follow up.

Parameter	Coefficient	95% CI	P value
Intercept	685,548	658,957 to 712,139	<0.001
TIME (years)	−10,704	−13,611 to −7,797	<0.001
HEMORRHAGE (1 = Yes)	3,321	−87,472 to 94,114	0.943
HEMORRHAGE x TIME	−11,529	−21,222 to −1,834	0.020

Legend:

Intercept = estimated number of retinal ganglion cells at baseline; TIME = duration of follow-up; HEMORRHAGE X TIME = interaction between the presence or absence of disc hemorrhage and the variable TIME; Coefficient = estimated value of each parameter; 95% CI = confidence interval.

Table 3 shows the results of a multivariable model including confounding variables. To investigate the effect of these variables on rates of change in estimated RGC counts, variable-time interaction terms (VARIABLE X TIME) were also included for each of these variables. The interaction term IOP X TIME was significant in the model (P = 0.033), indicating that eyes with higher IOP during follow-up had significantly faster rates of estimated RGC loss. However, there was no significant effect of age or CCT on rates of RGC loss, with the interaction terms AGE X TIME and CCT X TIME not significant in this sample (Table 3). There was also no significant effect of ancestry or gender on rates of RGC loss (P = 0.266 for ANCESTRY X TIME and P = 0.980 for GENDER X TIME).

**Table 3:** Results of the random coefficients multivariable model examining the association between estimated number of retinal ganglion cells (RGCs) and disc hemorrhages, including potentially confounding variables and their interactions with time.

Parameter	Coefficient	95% CI	P value
Intercept	683,257	660,261 to 706,254	<0.001
TIME (years)	−10,915	−13,821 to −8,009	<0.001
HEMORRHAGE (1 = Yes)	25,973	−52,748 to 104,694	0.518
HEMORRHAGE x TIME	−11,698	−21,287 to −2,109	0.017
Age	−6,413	−8,453 to −4,373	<0.001
Age X TIME	123	−126 to 372	0.332
Mean IOP	1,297	−205 to 2,800	0.091
Mean IOP x TIME	−579	−1,111 to −47	0.033
CCT	1,403	857 to 1,949	<0.001
CCT x TIME	−11	−81 to 59	0.758

Legend:

Intercept = estimated number of retinal ganglion cells at baseline; TIME = duration of follow-up; HEMORRHAGE X TIME = interaction between the presence or absence of disc hemorrhage and the variable TIME; Coefficient = estimated value of each parameter; 95% CI = confidence interval.

**Figure 2:**
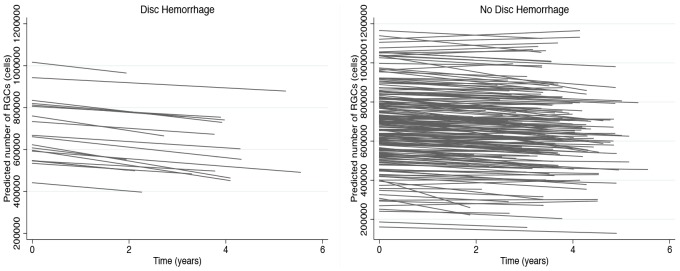
Change in estimated number of retinal ganglion cells (RGCs) over time in eyes with and without disc hemorrhages during follow up.

**Figure 3:**
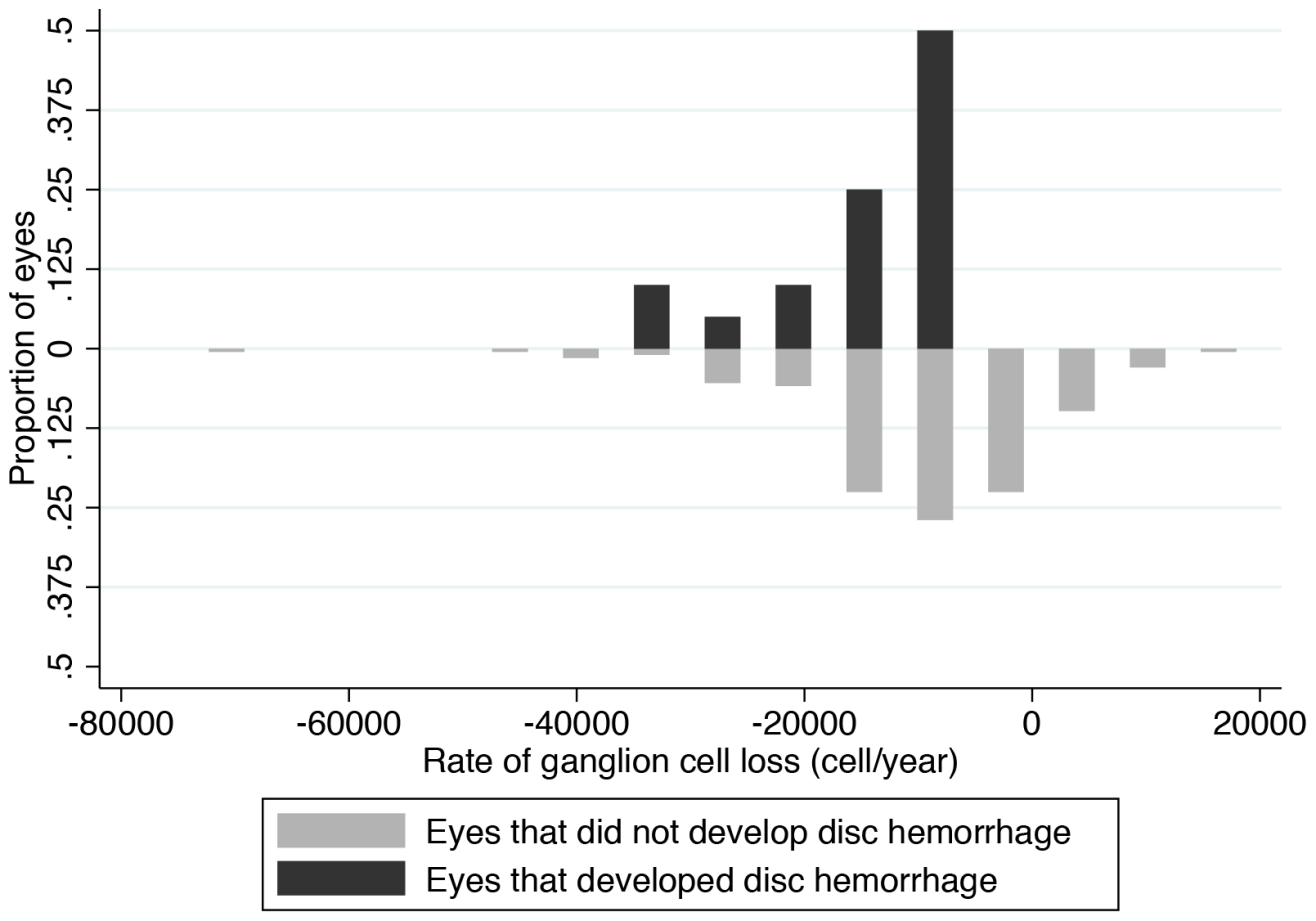
Histogram showing the distribution of slopes of change in estimated retinal ganglion cell (RGC) counts in eyes with and without disc hemorrhage during follow up.

**Figure 4:**
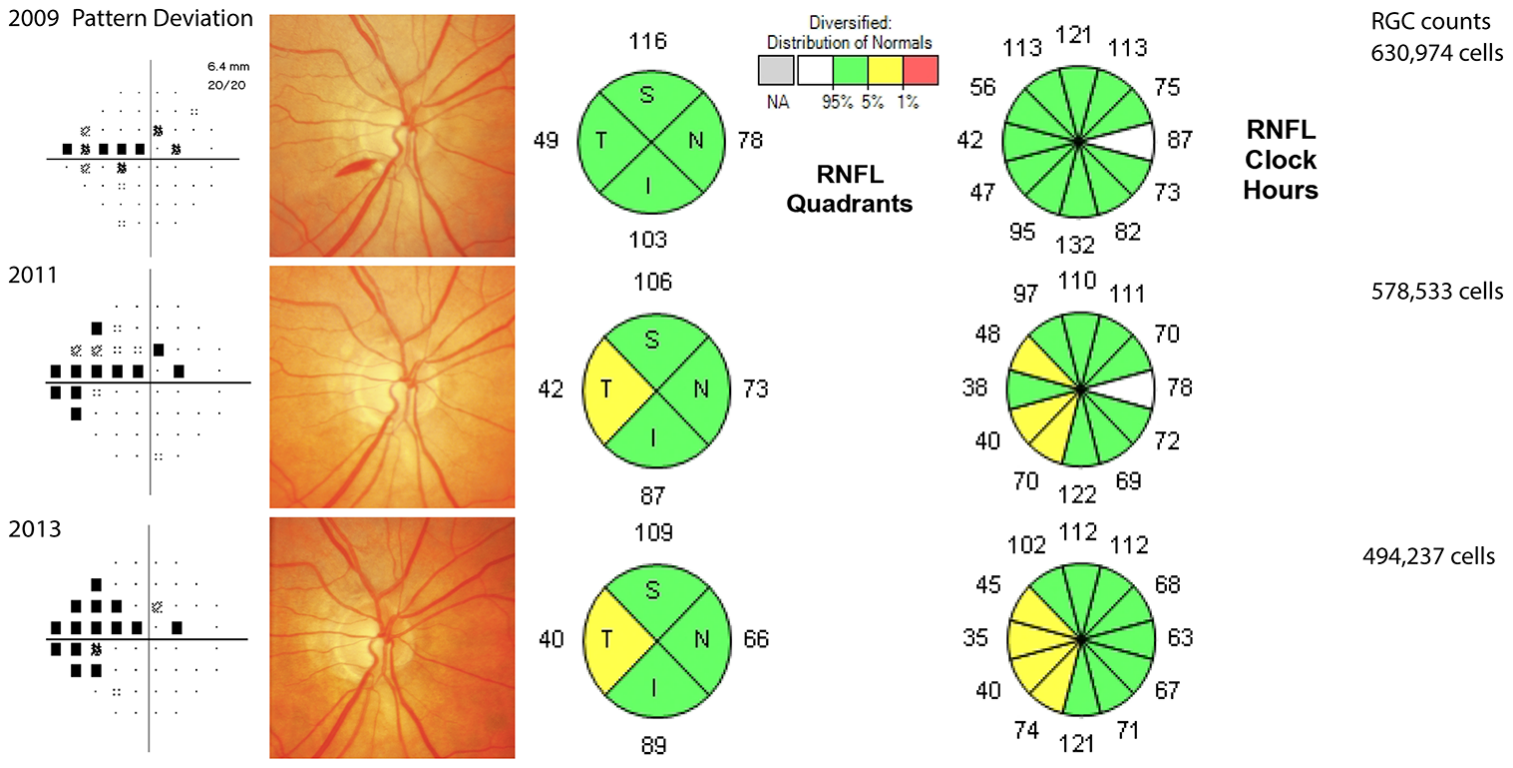
Example of an eye of a 77-year-old patient with a disc hemorrhage during follow up. Standard automated perimetry pattern deviation plot, optic disc photographs and optic coherence tomography results are shown at the time of disc hemorrhage and also of selected tests during follow-up. The estimated retinal ganglion cell count at baseline was 630,974 cells and decreased to 494,237 cells at the last follow up, with an estimated loss of 21,541 cells per year.

Figure 2 shows slopes of change over time in estimated RGC counts in individual eyes with and without disc hemorrhage included in the study and Figure 3 shows the distribution of estimated slopes of RGC loss in these eyes. Examples of eyes with and without optic disc hemorrhages are shown in Figure 4.

## Discussion

This study demonstrates that glaucomatous eyes with disc hemorrhages are likely to demonstrate faster rates of RGC loss compared to glaucomatous eyes without disc hemorrhages. This finding provides further evidence that disc hemorrhages are an important indicator of increased risk of progression in glaucoma. We found that eyes with optic disc hemorrhages during follow up lost on average an estimated 22,232 RGCs per year, a rate twice as fast as the 10,704 cells per year estimated loss in eyes in which a disc hemorrhage was not detected during follow up. In agreement with previous studies[8] [22]–[28], higher mean IOP during follow-up was also associated with faster rates of progression; even when accounting for IOP, however, the presence of a disc hemorrhage was still associated with a faster rate of RGC loss.

The present results are in agreement with previously published studies that have shown disc hemorrhages to be associated with structural and functional loss in glaucoma. For example, Medeiros and colleagues[8] have previously shown that rates of progressive visual field loss in eyes with optic disc hemorrhage were significantly faster than in eyes without disc hemorrhages. Furthermore, De Moraes and colleagues studied the relationship between optic disc progression and rates of visual field change in 389 treated glaucoma patients and recently reported disc hemorrhage to be the single most significant predictor for progressive visual field loss.[29] They found the presence of a disc hemorrhage to be a strong predictor of future visual field progression with an odds ratio of 5.38. Our study supports these previous conclusions, and also provides new information as we examined the relationship between disc hemorrhages and estimated changes in underlying neuronal loss in glaucoma. Importantly, the estimated RGC counts were derived from a combination of structural and functional information and these estimates have been shown to have better ability to detect and stage glaucomatous damage than isolated measures. [11], [12]

Previous studies have examined the spatial relationship between disc hemorrhages and structural and functional losses. [30]–[36] Although previous studies have not examined the association between RGC loss and disc hemorrhages, it is widely recognized that disc hemorrhages are commonly located at the border of or close to areas of retinal nerve fiber layer defects or neuroretinal rim loss. [30], [32]–[34] This suggests that the location of disc hemorrhages may provide information regarding the location of RGC loss. Although we did not examine localized rates of RGC loss, we found the commonest location for disc hemorrhages was the inferior temporal sector of the optic disc, in agreement with previous observations. In our sample, in 15 of 19 eyes (83%) the disc hemorrhage was located at the inferior temporal region of the optic disc or peripapillary RNFL.

Optic disc hemorrhages in glaucoma may be a marker of pressure-related glaucomatous damage. It has been proposed that serious consideration should be given as to advancing treatment if a disc hemorrhage is seen. [2], [37]–[39] Indeed a previous study has shown that lowering of IOP is likely to slow further visual field loss after a disc hemorrhage.[8] Although eyes with disc hemorrhages show faster rates of change on average compared to those without hemorrhages, it is important to emphasize that the rates of progression in eyes with disc hemorrhages can be widely variable. While some eyes develop fast progression, others continue to progress at a relatively slow rate despite the presence of a new hemorrhage. Therefore, the decision to intensify treatment in the presence of a disc hemorrhage should always take into account the risk of development of functional impairment or disability from the disease, as well as considerations about the patient's life expectancy and potential risks and side effects of treatment.

It is also important to emphasize that, although eyes with disc hemorrhages showed faster rates of estimated RGC loss compared to those without hemorrhages, a causal relationship between hemorrhages and progression cannot be established. It is possible that hemorrhages may be a marker for higher susceptibility to glaucoma progression, but without being in the causal pathway of neural damage. In this situation, ocular hypotensive treatments would still slow down the rate of progression in eyes with hemorrhages, as previously established, but they would not necessarily decrease the incidence of hemorrhages. In fact, results from the EMGT did not show a difference in the incidence of hemorrhages between treated and untreated groups followed over time.[6] A recent report examined rates of visual field progression before and after a disc hemorrhage.[9] Localized visual field loss was found to occur prior to disc hemorrhage and to continue after the event. This observation suggests that disc hemorrhages may be both a product of progressive glaucomatous damage and an indicator of possible future progression.

This study has limitations. First, empirically derived formulas were used to estimate the number of RGCs and the original formulas were derived from studies in a primate model of glaucoma and likely need refinement.[10] However, the formulas have been validated in multiple external human cohorts [11], [12], [17] and the RGC estimates obtained using these formulas are closely related to the findings of human histological studies. [10], [40]–[42] Another potential limitation is that some patients in the no hemorrhage group may have experienced a disc hemorrhage that went undetected during follow up. This is a potential problem as disc hemorrhages may resolve fairly quickly. Kitazawa and colleagues reported that disc hemorrhages are typically present for 4 weeks to 2 months before they resorb and disappear. [43] It is important to note that even if some patients without observable disc hemorrhage may have had undetected bleeding during follow-up, this would actually tend to decrease the difference between the two groups. Therefore, our results may be seen as conservative estimates of the differences in rates of change between the two groups. Moreover, evaluation of stereophotographs is a good method for detection of disc hemorrhages. In the OHTS 84% of patients with disc hemorrhages were identified only in photographs whereas only 16% were identified by both clinical examinations and photographs. [5] It should also be acknowledged that we did not examine the temporal relationship between the appearance of a disc hemorrhages and disease progression. Instead, rates of RGC loss were calculated for the entire follow up period and disc hemorrhages could have occurred at any time during follow up. This approach was used due to the relatively limited follow up time available with HDOCT measurements. Future studies should attempt to investigate the temporal relationship between optic disc hemorrhages, structural measurements and estimated rates of RGC loss in glaucoma.

In conclusion, this study indicates that glaucomatous eyes with disc hemorrhages during follow up are likely to show faster rates of RGC loss compared to glaucomatous eyes without disc hemorrhages. Our results provide further evidence that disc hemorrhages are an important clinical finding and should be considered as an indicator of increased risk for faster neural loss in glaucoma.
